# Rare Diseases Leading to Childhood Glaucoma: Epidemiology, Pathophysiogenesis, and Management

**DOI:** 10.1155/2015/781294

**Published:** 2015-09-16

**Authors:** Solmaz Abdolrahimzadeh, Valeria Fameli, Roberto Mollo, Maria Teresa Contestabile, Andrea Perdicchi, Santi Maria Recupero

**Affiliations:** ^1^Ophthalmology Unit, DAI Head/Neck, Umberto I Policlinic, University of Rome “Sapienza”, Viale del Policlinico 155, 00161 Rome, Italy; ^2^Ophthalmology Unit, Department of Sense Organs, University of Rome “Sapienza”, Viale del Policlinico 155, 00161 Rome, Italy; ^3^Ophthalmology Unit, St. Andrea Hospital, NESMOS Department, University of Rome “Sapienza”, via di Grottarossa 1035-1039, 00189 Rome, Italy

## Abstract

Noteworthy heterogeneity exists in the rare diseases associated with childhood glaucoma. Primary congenital glaucoma is mostly sporadic; however, 10% to 40% of cases are familial. CYP1B1 gene mutations seem to account for 87% of familial cases and 27% of sporadic cases. Childhood glaucoma is classified in primary and secondary congenital glaucoma, further divided as glaucoma arising in dysgenesis associated with neural crest anomalies, phakomatoses, metabolic disorders, mitotic diseases, congenital disorders, and acquired conditions. Neural crest alterations lead to the wide spectrum of iridocorneal trabeculodysgenesis. Systemic diseases associated with childhood glaucoma include the heterogenous group of phakomatoses where glaucoma is frequently encountered in the Sturge-Weber syndrome and its variants, in phakomatosis pigmentovascularis associated with oculodermal melanocytosis, and more rarely in neurofibromatosis type 1. Childhood glaucoma is also described in systemic disorders of mitotic and metabolic activity. Acquired secondary glaucoma has been associated with uveitis, trauma, drugs, and neoplastic diseases. A database research revealed reports of childhood glaucoma in rare diseases, which do not include glaucoma in their manifestation. These are otopalatodigital syndrome, complete androgen insensitivity, pseudotrisomy 13, Brachmann-de Lange syndrome, acrofrontofacionasal dysostosis, caudal regression syndrome, and Wolf-Hirschhorn syndrome.

## 1. Introduction

Childhood glaucoma is a rare disorder that occurs from birth until teenage years. It usually results in grave visual deterioration [1]. In the pediatric population approximately 8% of blindness has been attributed to the condition [2].

The European Glaucoma Society discriminates* primary congenital glaucoma* (birth to 2 years of age) and* early juvenile glaucoma* (2 years of age to puberty), due to incomplete development of trabecular meshwork, from* secondary childhood glaucoma* [3].

Secondary childhood glaucoma is defined as* associated with congenital ocular anomalies* such as conditions associated with mesodermal dysgenesis of the neural crest, phakomatoses characterized by hamartomas, metabolic disorders, mitotic disorders, and other congenital disorders and* associated with acquired conditions* such as tumors, uveitis, and trauma [3]. Finally, there are rare case reports of diseases that do not normally present glaucoma in their manifestation where the heterogeneity and sporadic nature of the cases make correlations with systemic syndromes and diseases complex and difficult. The purpose of this paper is to review the major conditions associated with secondary childhood glaucoma, providing epidemiological data and notions on pathophysiogenesis, and to assess rare diseases that usually do not include congenital glaucoma among their manifestations.

## 2. Epidemiology

### 2.1. Primary Congenital Glaucoma

The incidence of childhood glaucoma is 1 in 10,000 to 68,000 live births. A new case of primary congenital glaucoma is diagnosed about once every 5 years in a general ophthalmology center in North America or Western Europe [4–6]. The highest prevalence of childhood glaucoma has been recorded in the Middle East (1 : 2500) [7] and in Slovakia (1 : 1250) [8]. In previous epidemiological studies, the incidence of primary congenital glaucoma was reported as 1 in 10,0003 [9, 10] to 1 in 12,5005 in Western countries. A recent study reported the incidence as 3.31 in 100,000 (1/30,200) in Great Britain and 5.41 in 100,000 (1/18,500) in the Republic of Ireland [5]. Recently, the incidence of primary congenital glaucoma in Spain was reported as 2.85 per 100,000 (1 in 38,000). This is comparable to results in an Australian study where the incidence was 1 in 30,000 births [11]. The presence of consanguinity of parents can increase the incidence by 5 to 10 times [3]. In about 70% of cases, patients' both eyes are affected and it is more common among males [3].

Large epidemiological studies are difficult to carry out as childhood glaucoma presents so infrequently, even if it is the most common glaucoma in the pediatric population [12]. Moreover, since childhood glaucoma appears in the context of systemic diseases, most reports do not specifically describe ophthalmologic anomalies. Therefore, evaluation of the relationship between childhood glaucoma and rare diseases becomes difficult.  Table 1 is a classification of childhood congenital glaucoma.

### 2.2. Mesodermal Dysgeneses of the Neural Crest

The first group of rare systemic diseases leading to secondary childhood glaucoma are those associated with mesodermal dysgenesis of the neural crest [13]. The Axenfeld-Rieger syndrome prevalence is 1–9/1000 000 [14] and glaucoma has been observed in 50% of patients [13]. The prevalence of Peter's syndrome is beneath 1/1000000 [14]. Aniridia has an estimated prevalence between 1 : 40,000 and 1 : 100,000 [15] and the incidence of glaucoma in congenital forms ranges from 6% to 75% [16–18]. Marfan syndrome is a connective tissue disorder with autosomal dominant inheritance and a prevalence of 1 : 5000 [19]. In an international study including 1,000 patients with this syndrome, more than half presented eye manifestations such as myopia (53%), ectopia lentis (54%), and glaucoma (2%) [20]. Weill-Marchesani syndrome is a very rare systemic disease that implicates anomalies of connective tissue [21] and its prevalence has been estimated at 1 : 100,000 [22]. The main ocular anomalies of Weill-Marchesani syndrome are microspherophakia, ectopia lentis, glaucoma, and myopia. Microspherophakia occurs in 84% of cases, ectopia lentis in 73%, and glaucoma in 80%; severe myopia and cataract are also reported [21].

### 2.3. Phakomatoses

The second group of rare diseases linked with secondary childhood glaucoma are the phakomatoses characterized by hamartomas. Von Recklinghausen's diseases or neurofibromatosis type 1 is characterized by autosomic dominant inheritance and an incidence of about 1 in 3000 [23]. Glaucoma has been reported in 1/300 patients with this condition [24]. However, a study on 95 patients, with neurofibromatosis type 1, revealed that 23% of 56 patients presented orbitofacial involvement and had ipsilateral glaucoma [25].

Sturge-Weber syndrome has a frequency of approximately 1 in 50,000 births [26]. Glaucoma affects 50% to 70% of patients with this syndrome and its variants (such as Klippel-Trenaunay syndrome) and can be early- or late-onset depending on the age of manifestation [27]. In about 60% of cases the cause is due to anterior chamber abnormalities while in approximately 40% of cases the cause is raised episcleral venous pressure [28]. Oculodermal melanocytosis is a rare condition more frequently encountered in the Asian population with a prevalence of 0.014%–0.034% [29]. Glaucoma is found in about 10% of patients and the pathogenesis may be congenital, developmental or associated with the diffuse characteristic oculodermal hyperpigmentation of the disease which leads to melanocytic glaucoma [30–32]. Phakomatosis pigmentovascularis is a rare combination of oculodermal melanocytosis and Sturge-Weber syndrome (or Klippel-Trenaunay syndrome). In a review of the literature on 281 Asian patients with oculodermal melanocytosis, 9 patients had phakomatosis pigmentovascularis and glaucoma was present in all cases [30].

### 2.4. Metabolic Disorders

The metabolic disorders leading to childhood glaucoma include mucopolysaccharidoses, Lowe's syndrome, and classical homocystinuria. Lowe's syndrome (oculocerebrorenal syndrome) has a prevalence of 1 in 500,000 [33]. Several cases of secondary congenital glaucoma associated with this disease have been reported; Walton et al. reviewed 7 patients and found that 71% of patients had glaucoma [34]. Classical homocystinuria is caused by cystathionine b-synthase (CbS) deficiency [35–37]. The detection rate of CbS deficiency is 1 in 344,000 [38]. Ectopia lentis, which is often associated with classical homocystinuria [39, 40], occurs in 85% of classical homocystinuria [39, 41, 42]. In a report on 294 patients with mucopolysaccharidoses, 27 eyes of 14 patients had glaucoma and the prevalence of glaucoma was reported from 2.1% to 12.5% [43].

### 2.5. Mitotic Disorders

The group of mitotic disorders leading to childhood glaucoma includes juvenile xanthogranuloma, the prevalence of which is unknown. It occurs in the pediatric age and 10% of patients present ocular anomalies with occasional glaucoma [44].

### 2.6. Other Congenital Conditions

The prevalence of trisomy 13 or Patau's syndrome is 1/6500 where glaucoma has been reported among the ocular complications [45].

In a study of 62 cases of persistent hyperplastic primary vitreous, Haddad et al. reported 26 patients where other ocular abnormalities such as leukocoria, microphthalmia, cataract, retinal detachment, glaucoma, and phthisis bulbi were observed [46].

Five to twenty percent of pediatric blindness is attributable to congenital cataract, which occurs in 3-4 per 10 000 births [47]. Although new surgical techniques have been introduced, the incidence of secondary glaucoma following cataract operations in the pediatric age is still high, varying from 6% to 26% [48]. If the surgical procedure is performed before the 9th month of life, the incidence of glaucoma may rise to 50% [48, 49]. Rubinstein-Taybi syndrome as well as congenital rubella appears among congenital disorders associated with glaucoma. Rubinstein-Taybi syndrome has an incidence of 1 in 100 000 newborns [50]. van Genderen et al. reviewed 207 patients with Rubinstein-Taybi syndrome, and 117 had ocular disorders. Congenital glaucoma was described in 31 cases [51]. Epidemiological assessments of ocular disease in large populations of congenital rubella are few. In France just 21 cases of rubella during pregnancy were reported in 2001 and only one child presented congenital rubella syndrome at birth [52]. Various ocular disorders such as retinopathy, cataract, and glaucoma may occur in this condition [53]. In a prospective study by O'Neill in 34 patients with rubella, glaucoma was observed in 29% (11 eyes) and was in the context of microphthalmic eyes in 4 patients [54].  Table 2 is a summary of the epidemiology and percentage of glaucoma in rare diseases leading to secondary childhood glaucoma.

### 2.7. Rare Diseases

Cases reports of congenital glaucoma have been reported in the context of rare systemic syndromes such as otopalatodigital syndrome type II (OPD II) [55], complete androgen insensitivity [56], pseudotrisomy 13 [57], Brachmann-de Lange syndrome [58], autosomal recessive acrofrontofacionasal dysostosis [59], caudal regression syndrome [60], and Wolf-Hirschhorn syndrome [61] (Table 3).

## 3. Genetics and Pathophysiology

### 3.1. Primary Congenital Glaucoma

While most cases of primary congenital glaucoma are sporadic, 10% to 40% are familial and are often associated with consanguinity [62]. Familial forms have autosomal recessive inheritance with variable expression in 40 to 100% of cases [63]. However, the autosomal recessive inheritance modality has been challenged due to the discrepancy between gender distribution, transmission of childhood glaucoma to offspring, and lower predicted afflicted siblings in familial cases [63]. Linkage studies confirm the heterogeneity of primary congenital glaucoma, so these discrepancies can be explained [63, 64].

Primary congenital glaucoma has been associated with 3 chromosomal loci: chromosomes 2p21 (GLC3A), 1p36 (GLC3B), and 14q24 (GLC3C) [62, 63, 65, 66].

The mutation of the CYP1B1 gene is involved in 87% of familial and 27% of sporadic cases of congenital glaucoma [63]. Currently 147 mutations of this gene have been found, including point missense, nonsense, frameshift, terminator mutations, deletion, and insertion with significant heterogeneity [67, 68]. CYP1B1 is a drug-metabolizing enzyme of the cytochrome p450 gene family. It is expressed in a large spectrum of tissues including the iris, trabecular meshwork, ciliary body, and anterior uveal tract of the eye [69]. Ocular development is associated with the signalling molecule, metabolized by the gene product, but the procedure still needs clarification [68]. Primary congenital glaucoma is not related to juvenile or adult onset open angle glaucoma linked to the MYOC (TIGR) gene on chromosome 1q25 at locus GLCIA [70], but in some cases a mutation in the CYP1B1 has been found [71].

Alteration of the trabecular meshwork leading to a reduction of aqueous outflow results in childhood glaucoma. It may be a consequence of developmental defects or trabecular structure immaturity [72, 73]. There is no documentation of imperforate Barkan's membrane over the anterior chamber angle, although the trabecular meshwork anomaly was initially described as a “clothed membrane with a shagreened surface” [74–76]. The histopathology of childhood glaucoma is characterized by five different features. One such aspect is an anterior insertion of the iris and ciliary body; thus, the trabecular meshwork is inconsistently exposed to aqueous deflux [76]. The overlap between the ciliary body in its anterior section and the trabecular meshwork in its posterior part leads to poor development of the angle recess. The ciliary muscle, with its longitudinal fibers, passes the internal tip of a poorly developed scleral spur; thus, it obtrudes in the corneoscleral meshwork [77]. The second feature consists in taut and thickened trabecular beams in the meshwork [62]. A third characteristic is a less porous and thicker juxtacanalicular meshwork [76, 78, 79] possibly due to scarce differentiation [73]. Fewer vacuoles in Schlemm's canal endothelium have been described in eyes affected by congenital glaucoma; this may be either an artefact or a consequence of the decrease of aqueous flow [76, 78, 79]. Finally, agenesis of Schlemm's canal has been described; it occurs rarely and probably indicates more severe maldevelopment [73, 80].

Similarity of the angle histopathology between some cases of secondary childhood glaucoma and primary congenital glaucoma suggests that a common pathophysiogenetic mechanism related to angle development may exist.

### 3.2. Mesodermal Dysgeneses of the Neural Crest

Secondary childhood glaucoma in conjunction with ocular or systemic disorders can be gathered in different subgroups according to their etiology. It can be associated with mesodermal dysgenesis of the neural crest. Several syndromes result from an abnormal development of neuroectodermal cells [13]. In the eye, anterior chamber development follows the migration of neural crest cells forming keratocytes, corneal endothelium, iris stroma, melanocytes, trabecular meshwork, and juxtacanalicular tissue [13]. The gamma of anterior segment dysgeneses, or Axenfeld-Rieger syndrome, is not linked to genetic factors in congenital glaucoma. Mutations in the PITX2 and FOXC1 transcription factor genes account for anterior segment dysgeneses, which are autosomal dominant disorders [81, 82]. A developmental arrest or an abnormal migration of neural crest cells, occurring late in gestation, may lead to chamber malformation resulting in Axenfeld-Rieger anomaly, Peter's anomaly, aniridia or iris hypoplasia, sclerocornea, megalocornea, and congenital glaucoma [13, 83]. These anomalies together with the Marfan and Weil-Marchesani syndromes are linked to glaucoma because of an abnormal anterior chamber angle that effects the drainage of aqueous humour [13].

### 3.3. Phakomatoses

Several cases of secondary congenital glaucoma have been reported in association with the phakomatoses and hamartomas. Neurofibromatosis type 1, encephalotrigeminal angiomatosis (Sturge-Weber syndrome and variants such as Klippel-Trenaunay syndrome), retinal and cerebellar angiomatosis, and oculodermal melanocytosis are included among these multisystem disorders [3, 84]. Glaucoma onset is seldom encountered in neurofibromatosis type 1 and the pathophysiological mechanisms reported are infiltration of the anterior chamber by neurofibromas, Lisch nodules in the chamber angle leading to secondary angle closure, increase in thickness of the ciliary body and choroid, and developmental angle abnormalities [85–88]. Secondary childhood glaucoma is frequently associated with the Sturge-Weber and Klippel-Trenaunay syndromes and with phakomatosis pigmentovascularis which have the facial naevus flammeus in common. This is due to anterior chamber malformation or raised episcleral venous pressure [30, 89–91]. In phakomatosis pigmentovascularis glaucoma can also be due to an increase in melanocytes which clog the angle. Glaucoma is not strictly part of the manifestations of the other phakomatoses, namely, Von Hippel-Lindau disease, which is characterized by retinal angiomatosis and sclerosis tuberous complex characterized by retinal hamartomas. In these diseases glaucoma has only been reported among the complications of retinal detachment [92].

### 3.4. Metabolic Disorders

Congenital glaucoma may secondarily occur in the context of metabolic disorders such as mucopolysaccharidoses, Lowe's syndrome, and hyperhomocysteinemia [3]. In these diseases the incidence of secondary glaucoma is attributed to both an anomalous structure of the anterior chamber and engorgement of the trabecular meshwork by extracellular material [3, 29, 84, 93, 94]. The mucopolysaccharidoses are inherited conditions with intra- and extracellular accumulation of glycosaminoglycans (GAG). All subtypes show ocular disorders among which are opacification of the cornea, retinal pathology, and secondary glaucoma [95]. Narrowing of the angle and obstruction of trabecular outflow are secondary to an accumulation of GAG within trabecular cells. [95].

Another condition in this group of metabolic disorders is Lowe's syndrome (oculocerebrorenal syndrome), a rare X-linked, recessively transmitted disease [28] which is characterized by a mutation of phosphatidylinositol 4,5 biphosphate 5-phosphatase, resulting in intracellular accumulation of its PIP2 substrate leading to systemic anomalies involving the kidneys, central nervous system, and the eyes [96]. Classical homocystinuria follows an autosomal recessive mode of inheritance and is a disorder of methionine metabolism [97] due to deficiency of cystathionine b-synthase (CbS) [30–32]. Ectopia lentis represents the most common clinical anomaly leading to diagnosis in most cases [34, 35]. Anterior dislocation of the lens sometimes leads to acute pupillary block with a resulting secondary glaucoma [34].

### 3.5. Mitotic Disorders

Mitotic disorders such as juvenile xanthogranuloma may present secondary congenital glaucoma as their ocular manifestation [84]. Juvenile xanthogranuloma is due to histiocytic abnormalities that rarely occur in the pediatric age [98, 99]. This disease can damage various ocular structures, but the iris is most frequently affected [100]. The onset of secondary glaucoma is frequently related to the presence of iris nodules that, due to their vascular nature, may bleed causing hyphema [101].

### 3.6. Other Congenital Conditions

Secondary childhood glaucoma can also be associated with other disorders such as persistent hyperplastic primary vitreous, congenital cataract, Patau's syndrome, Rubinstein-Taybi syndrome, or congenital rubella [84, 102, 103]. Secondary childhood glaucoma is often associated with congenital cataract and it may occur as postoperative complication of pediatric cataract surgery [48]. Different ocular anomalies are associated with Rubinstein-Taybi syndrome, which results from microdeletions at chromosome 16p13.3 or from mutations in the gene for the CREB binding protein (CBP), located at 16p13.3 [104, 105].

### 3.7. Acquired Conditions

Uveitis and steroid induced glaucoma are among the major acquired conditions associated with childhood glaucoma. Trauma induced glaucoma can be caused by hyphema, angle recession, and ectopia lentis. Ocular or orbital, benign or malignant, tumors and retinopathy of prematurity can also be a cause. Retinopathies and some drugs can also cause secondary glaucoma and the pathophysiogenetic mechanism is specific to each condition [106–108].

### 3.8. Rare Diseases

There are some rare diseases, which do not normally present glaucoma in their manifestation.

Kondoh et al. published a case of bilateral glaucoma in a male child presenting otopalatodigital syndrome type II (OPD II) related to a mutation of the gene for filamin A (FLNA). Performing a sequence analysis of the FLNA gene, a missense C to T mutation at position 588, resulting in an Arg196Trp change in the filamin A protein, was found [55]. FLNA gene encodes an actin-binding protein and mutations of the FLNA gene have been found in OPD II, Melnick-Needles syndrome, or frontometaphyseal dysplasia. The association between OPD II, congenital heart defects, and ocular disorder, as presented by Kondoh et al., has not been previously reported. Therefore, additional factors are probably involved in the pathogenetic mechanism behind the OPD-group alterations [55].

Gad et al. reported an association of complete androgen insensitivity with hypertrophic pyloric stenosis and congenital glaucoma in an Egyptian newborn. Performing a sequence of the five exons of the 5a-reductase type 2 gene, no evidence of mutation was obtained while a C to T mutation, which resulted in substitution of the phenylalanine residue by a leucine at position 804, was identified in exon 6. The father's family had a history of glaucoma, ruling out the causative effect of the receptor gene in the pathogenesis of glaucoma and exon 6 of the androgen receptor gene of the mother was normal; therefore, the mutation was considered de novo. It is possible that defective androgen action determined the congenital glaucoma. Moreover, it is interesting to note that CYP1B1 gene, which is the most common early-onset glaucoma gene, is related to steroids. The CYP1B1 gene lies next to the SRD5A2 gene on 2p21 and both the androgen receptor and SRD5A2 genes are expressed in eye structures. However, the consequences of defective signalling pathways on the structure and function of ocular tissues and the correlated gene are still unknown [56].

Sandal et al. reported the only case of congenital glaucoma in a female infant affected by pseudotrisomy 13. Detailed genetic examination, conventional karyotype, and microarray studies are necessary to assess anomalies that are not usually related to this syndrome [57].

Barry Lee et al. first reported a case of buphthalmos and childhood glaucoma associated with Brachmann-de Lange syndrome. Chromosome 3q16 is probably related to Brachmann-de Lange syndrome, while some forms of heritable childhood glaucoma have been mapped to chromosomes 1 and 2. Further genetic studies could be useful to assess a correlation between these disorders [58].

Chaabouni et al. reported the first case of congenital glaucoma in a patient who suffered from acrofrontofacionasal dysostosis with genitourinary abnormalities [59]. As far as we know, this is one of just three case reports in this condition, which has autosomic recessive transmission. The paucity of cases precludes our understanding of the ophthalmological anomalies. Guirgis et al. first reported a case of childhood glaucoma associated with caudal regression syndrome in a 10-week-old infant. The child also had punctal atresia and significant myopia. Embryonic defect of caudal mesodermal development seems to be the cause of caudal regression syndrome [109]. However, goniodysgenesis, caused by neural crest alterations, probably led to childhood glaucoma. A faulty development of surface ectoderm is related to punctual atresia. The embryopathic nature of both caudal regression syndrome and childhood glaucoma, as well as their low incidence, implies that there might be a common cause rather than a coincidence. Furthermore, concomitant punctual atresia validates the notion of embryopathic linkage [110].

Dickmann et al. tried to analyze the relationship between Wolf-Hirschhorn syndrome and ocular defects, specifically to correlate ocular findings with the extension of deletion on chromosome 4, so they investigated a population of 10 patients affected by Wolf-Hirschhorn syndrome [61]. This condition is a genetic disorder that occurs as a consequence of partial deletion of the short arm of chromosome 4; the proximal breakpoint occurs between 4p15.1 and 4p16.3, and the smallest and largest deletion sizes recorded were 2.6 Mb and 20 Mb, respectively [61, 111, 112]. Ocular anomalies appeared in all patients, but just one patient was affected by childhood glaucoma. Comparing genotype with ocular findings, severe ocular abnormalities were associated with large 4p deletions while mild ocular disorders were independent of the deletion size [61]. The only case of childhood glaucoma was observed at birth; it appeared with concomitant corneal clouding, iris coloboma, and cataract. It was associated with a large 4p deletion [61, 113].

## 4. Management

The signs and symptoms of childhood glaucoma are closely linked to the age of onset and the gravity of the disease; however, some children are asymptomatic. Corneal alterations can be edema, Descemet's membrane breaks, and opacification. This leads to the typical manifestations of photophobia, epiphora, and consequent blepharospasm. In some cases a misinterpretation of these symptoms delays diagnosis [62]. Improved instrumental technology with optical coherence tomography and ultrabiomicroscopy can provide details on the anterior chamber and ciliary body which is helpful towards diagnosis [114, 115]. The management of childhood glaucoma is also facilitated by new instrumentation to measure intraocular pressure and visual field defects [116–119].

Any type of glaucoma leads to irreversible vision loss in the long term; therefore, the goal of both medical and surgical management is to prevent visual deterioration [62]. Treatment should be carefully tailored, aiming to control intraocular pressure in the course of time. Surgical procedures represent the mainstay of therapy for childhood glaucoma. Although medical therapy alone is rarely effective, it plays an important role as adjunctive treatment to surgery; moreover, it can also be used temporarily before surgery to clear the cornea [62]. Ophthalmologists who use medication should be aware of different risk and benefit profiles in infants and children compared with adults. Allergic reactions and ocular surface toxicity may complicate repeated topical treatment initiated at an early age in childhood [62].

### 4.1. Medical Treatment

Beta-blockers are usually well tolerated and severe systemic complications are rare in pediatric patients treated with timolol, but bradycardia and bronchospasm can occur especially with the concentration of 0.5%. Cautious initial treatment with low concentrations of timolol is wise. Punctal occlusion after topical timolol determines a decrement of beta-blocker plasma levels, so the use of punctual occlusion after every drop application is advised [62].

Oral carbonic anhydrase inhibitors represent the most efficacious drugs in the reduction of intraocular pressure in childhood glaucoma. Nevertheless it is important to be aware that these can cause decreased appetite and hyperpnea from renal acidosis, dehydration, chronic fatigue, and failure to grow, so prolonged use should be avoided [62]. Topical carbonic anhydrase inhibitors such as dorzolamide and brinzolamide represent a valid alternative to oral administration with less risk of systemic side effects. They reduce intraocular pressure by 25% [120, 121]. Carbonic anhydrase inhibitors and beta-blockers are both suppressors of aqueous production but are used in conjunction in order to yield additive benefit [122]. Brimonidine is a selective alpha-agonist, which reduces intraocular pressure increasing uveoscleral outflow and decreasing aqueous production [123]. Since brimonidine crosses the blood brain barrier, it can lead to nervous systemic toxicity [124]. Brimonidine topical therapy has also been reported to induce bradycardia, hypotension, hypotonia, hypothermia, apnea, and unresponsiveness in children [125–128].

Prostaglandin analogues such as bimatoprost, travoprost, and latanoprost are not effective in pediatric patients. Although in some cases of children affected by primary juvenile glaucoma and glaucoma related to Sturge-Weber syndrome a significant intraocular pressure reduction was found [129, 130], further research is necessary in order to assess the performance of prostaglandins in the treatment of childhood glaucoma.

Miotics such as pilocarpine are not effective for childhood glaucoma. In older children, symptoms of spasm of the ciliary muscle and blurring of vision from induced myopia occur. They can be used for aphakic glaucoma and are adoperated before goniosurgery [62]. Most often childhood glaucoma is better managed surgically rather than medically [62].

### 4.2. Surgical Treatment

Surgical treatment can be separated into procedures that improve aqueous outflow through the drainage channels (goniosurgery such as goniotomy and trabeculotomy), treatments that by destroying the ciliary body reduce aqueous humor production (cyclodestruction), or surgical procedures that drain aqueous through an alternative way (trabeculectomy, glaucoma drainage devices) [131].

While for primary congenital glaucoma goniotomy represents the procedure of choice, this indication is less certain for other forms of childhood glaucoma [62]. An alternative surgical treatment is trabeculotomy [62]; it appears as a more appropriate choice especially when a cloudy cornea prevents a comfortable visualization of the angle [62]. Filtering surgery may be indicated if goniosurgery procedures are unsuccessful [3]. Trabeculectomy is indicated if an unacceptable elevation of intraocular pressure persists after an adequate number of angle incision procedures [62]. Implant surgery represents a valid alternative for patients with conditions proven to be refractory to goniosurgery or filtering surgery, who are poor candidates to these procedures, or for whom these treatments have been revealed unsuccessful [62]. Long tube drainage device surgery is needed in severe cases of primary congenital glaucoma and, sometimes, in secondary glaucomas [3].

Cyclodestructive procedures can be defined as an intermediate or an additional procedure, useful when primary trabecular surgery has failed [3]. Cyclocryotherapy or both transscleral and endoscopic diode laser cyclophotocoagulation are used for the ablation of ciliary epithelium. The surgeon must consider the anatomic improvement, the long-term visual benefit, and the patient's expectations for the affected eye when making the choice for management strategies [62].

## 5. Conclusions

There is a wide array of systemic diseases associated with childhood glaucoma. Some conditions like dysgenesis of the anterior chamber associated with neural crest disorders and the phakomatoses are better known. However, there are rare diseases which are occasionally associated with glaucoma. Since the examination of pediatric patients is not always straightforward, ophthalmologists should be updated on the available literature and should be well aware of the conditions, which may lead to visual deterioration due to glaucoma. Treatment should be carefully tailored, in order to control intraocular pressure in the long term. Surgical treatment is the mainstay of management and medical therapy has an adjunctive role. Furthermore, medication should be carefully monitored due to different risk and benefit profiles in infants and children compared with adults. A multidisciplinary approach to pediatric patients with rare multisystem diseases is highly advisable in both the diagnosis and management of the conditions.

## Figures and Tables

**Table 1:** Classification of childhood glaucoma.

Primary congenital glaucoma (trabeculodysgenesis)	

Secondary childhood glaucoma	

Mesodermal dysgeneses of neural crest	Iridocorneal trabeculodysgenesis (i) Aniridia (ii) Axenfeld-Rieger's anomaly (syndrome if systemic associations) (iii) Peter's anomaly (syndrome if systemic associations) (iv) Marfan's syndrome (v) Weill-Marchesani's syndrome

	Von Recklinghausen's syndrome
Phakomatoses	Encephalotrigeminal angiomatosis (Sturge-Weber syndrome and variants such as Klippel-Trenaunay syndrome)
	Oculodermal melanocytosis

	Lowe's syndrome
Metabolic disorders	Homocystinuria
	Mucopolysaccharidoses

Mitotic disorders	Juvenile xanthogranuloma

	Trisomy 13 (Patau's syndrome)
	Persistent hyperplastic primary vitreous
Other congenital disorders	Congenital cataract: (i) In phakic eyes (ii) In aphakic eyes after early surgery
	Rubinstein-Taybi syndrome
	Congenital rubella

	Uveitis
	Trauma (hyphema, angle recession, and ectopia lentis)
Glaucoma associated with acquired conditions	Steroid or drug induced
	Tumors (benign/malignant, ocular/orbital)
	Retinopathy of prematurity

**Table 2:** Epidemiology and percentage of glaucoma in rare diseases leading to childhood glaucoma.

Primary congenital glaucoma 1 in 10,000 to 68,000			

Secondary childhood glaucoma			
Disease		Prevalence of disease	Percentage of glaucoma in disease

Mesodermal dysgeneses of neural crest	Iridocorneal trabeculodysgenesis		
	(i) Aniridia	From 1/40,000 to 1/100,000	6–75% [16–18, 24]
	(ii) Axenfeld-Rieger's anomaly (syndrome if systemic associations)	1–9/1000000	50% [13]
	(iii) Peter's anomaly (syndrome if systemic associations)	1/1000000	Not available
	(iv) Marfan's syndrome	1/5000	2% [20]
	(v) Weill-Marchesani syndrome	1/100000	80% [21]

	Von Recklinghausen's syndrome	1/3000	1% [17, 24], 23% of patients with ipsilateral craniofacial anomalies [25]
Phakomatoses	Encephalotrigeminal angiomatosis (Sturge-Weber syndrome and variants such as Klippel-Trenaunay syndrome)	1/50000	50–70% [27] (100% incidence in cases associated with oculodermal melanocytosis [32])
	Oculodermal melanocytosis	14/100000 to 34/100000	10% [29, 32]

	Lowe's syndrome	1/500000	71% [34]
Metabolic disorders	Homocystinuria	1/344000	85% of cases with ectopia lentis [38, 39, 41, 42]
	Mucopolysaccharidoses	1/25000	2.1% to 12.5% [43, 95]

Mitotic disorders	Juvenile xanthogranuloma	Unknown	10% of patients present ocular anomalies with occasional glaucoma [44]

Other congenital disorders	Trisomy 13 (Patau's syndrome)	1/6500	Not available
	Persistent hyperplastic primary vitreous	Unknown	31% of patients present ocular abnormalities with occasional glaucoma [46]
	Congenital cataract	1–6/10000	6–26% following cataract surgery [48]
	Rubinstein-Taybi syndrome	Unknown	15% [51]
	Congenital rubella	Unknown	29% [54]

**Table 3:** Epidemiology and glaucoma in rare diseases that do not usually include congenital glaucoma among their manifestations.

Secondary glaucoma		
Disease	Prevalence of disease	Percentage of glaucoma in the disease
Rare diseases		
Otopalatodigital syndrome type II	Unknown	One case [55]
Complete androgen insensitivity	1–9/1000000	One case [56]
Pseudotrisomy 13	Unknown	One case [57]
Brachmann-de Lange syndrome	1–9/100000	One case [58]
Acrofrontofacionasal dysostosis with genitourinary abnormalities	Unknown	One case [59]
Wolf-Hirschhorn syndrome	1–9/1000000	One case [61]
